# Effect of Surrogate Aggregates on the Thermal Conductivity of Concrete at Ambient and Elevated Temperatures

**DOI:** 10.1155/2014/939632

**Published:** 2014-02-13

**Authors:** Tae Sup Yun, Yeon Jong Jeong, Kwang-Soo Youm

**Affiliations:** ^1^Department of Civil and Environmental Engineering, Yonsei University, Yonsei-ro 50, Seodaemun-gu, Seoul 120-749, Republic of Korea; ^2^GS E&C Research Institute, Deokseong-ri, Cheoin-gu, Yongin-si, Gyeonggi-do 449-831, Republic of Korea

## Abstract

The accurate assessment of the thermal conductivity of concretes is an important part of building design in terms of thermal efficiency and thermal performance of materials at various temperatures. We present an experimental assessment of the thermal conductivity of five thermally insulated concrete specimens made using lightweight aggregates and glass bubbles in place of normal aggregates. Four different measurement methods are used to assess the reliability of the thermal data and to evaluate the effects of the various sensor types. The concrete specimens are also assessed at every 100°C during heating to ~800°C. Normal concrete is shown to have a thermal conductivity of ~2.25 W m^−1^ K^−1^. The surrogate aggregates effectively reduce the conductivity to ~1.25 W m^−1^ K^−1^ at room temperature. The aggregate size is shown not to affect thermal conduction: fine and coarse aggregates each lead to similar results. Surface contact methods of assessment tend to underestimate thermal conductivity, presumably owing to high thermal resistance between the transducers and the specimens. Thermogravimetric analysis shows that the stages of mass loss of the cement paste correspond to the evolution of thermal conductivity upon heating.

## 1. Introduction

New Korean energy-saving design standards for new buildings and houses effective from September 2013 seek to improve the energy efficiency of residential and office buildings that occupies 19.6% of the total energy consumption in 2007 [1, 2]. They aim to reduce the yearly household energy consumption for heating from its 2005 level of 120 kW h m^−2^ to below 30 kW h m^−2^ by 2017. This reduction is sought by having newly constructed houses contain more than 200 mm of polystyrene insulation or thicker concrete walls [1], measures which had previously been deemed too costly [3]. The use of inexpensive floor heating and internal insulation in the quickly built high-rise housing of Korea erected since the 1980s has resulted in formation of surface condensation and mold due to the temperature differential between the concrete walls and the internal insulation board.

External insulation could remedy this problem, but its installation would be costly and time consuming and may be hindered by legal regulations. The development of concrete with high thermal resistance is possibly a more practicable alternative. The thermal conductivity of concretes can be easily reduced by replacing one or more of its constituents with thermally insulating materials, such as lightweight coarse aggregates or glass bubbles [4]. Lightweight aggregates have been used, for example, in residential buildings in Japan, saving 20% of the heating energy consumption to maintain *∼*20°C room temperature compared with those of normal concrete [5]. Glass bubbles have also been widely used as thermal insulation in the manufacture of insulated pipes and heat-reflective paints [6]. Concretes, as complex mixtures of varying composition, can exhibit a wide range of thermal conductivities (i.e., 0.6~3.6 W m^−1^ K^−1^) depending on the aggregates used and the moisture conditions and also on the temperature range and method of testing [7–9]. The assessment of the thermal conductivity of concretes mixed with different synthetic materials and its variation at elevated temperature is challenging and more complicated than the assessment of normal concrete. Therefore, the development of methods to estimate accurately the thermal conductivity at different temperatures of concrete with normal or lightweight aggregate (LWA) is a crucial part of the design of thermally efficient infrastructure.

Previous experimental and numerical investigations have reported the thermal properties (e.g., thermal conductivity, specific heat, and thermal strain) of structural concrete and thermally insulated concrete containing LWA and additives such as fiber, recycled glass, and metakaolin at ambient and elevated temperatures [10–13]. The density and thermal conductivity of concrete often decrease upon heating. However, the evolution during heating of the microstructure of cement paste has not been sufficiently analyzed in concretes with either normal or lightweight aggregates. The role of lightweight aggregates and other additives also remains to be fully elucidated. Moreover, the reliability of thermal conductivity measurement depends not only upon the measurement method at either a steady state or during transient states but also on the transducer type (e.g., hot-guarded plate, hot box, and thermal needle probes) [4, 9, 14, 15]. The most important microstructural components of hydrated cement paste are calcium silicate hydrates (C–S–H), which make up to 67% of the hydration products, and calcium hydroxide [16]. These components determine the mechanical properties of the paste [17–19]. Dehydration of the calcium silicate hydrates and the dehydroxylation of the calcium hydroxide account for the mass loss observed during heating. The relationship between the thermal conductivity and the mass loss of the microstructural components of hydrated cement paste has not been clearly determined [19, 20].

This work presents an investigation of the thermal conductivity of various thermally insulated concretes. Samples containing different aggregates and glass bubbles are compared at ambient and elevated temperatures. A reference sample containing normal aggregate is compared against five different thermally insulated concrete specimens. The roles of the surrogate aggregates are explored by measuring the thermal conductivity of the samples using four different test methods: two that employ embedded probes (thermal needle probe and plane-source heating) and two that use contact hot-wire methods. One of the hot-wire methods is the ASTM C1113 standard method for the estimation of temperature-dependent thermal conductivity [21]. The effect of fine and coarse aggregates on the thermal conductivity is also evaluated. Thermogravimetric analysis (TGA) is used to compare the weight loss sequence during heating with the corresponding evolution of thermal conductivity. The relationship between the microstructural compositions of the cement pastes and their thermal conductivities is then evaluated.

## 2. Materials and Methods

### 2.1. Materials

Various combinations of ordinary Portland cement (ASTM Type I), fine aggregate, normal coarse aggregate, two types of lightweight coarse aggregates, and glass bubbles are used to fabricate the test specimens. The fine and course aggregates originate from crushed rocks of similar origin: they share the same mineralogy; only the grain size differs (Korea lacks a distinct natural source of fine aggregates such as cleaned coastal sand). Micrometer-sized glass bubbles (3 M, Ltd.) are tested as a partial replacement for the coarse aggregate and to create artificial pore spaces in the concretes. Two types of LWA (Argex, from Argex NV, Ltd., and Asanolite, from Taiheiyo Cement, Ltd.) are tested as replacements for the remaining coarse aggregate. The physical properties of the various aggregates and glass bubbles are listed in Table 1.

### 2.2. Specimen Preparation

Thermally insulated concretes are prepared by replacing the coarse aggregate with the glass bubbles and the lightweight aggregates. Detailed mixing proportions are listed in Table 2. K denotes a specimen with glass bubbles; the appended number represents the volume fraction of glass bubbles added with respect to the total aggregate volume. The effects of aggregate size and volumetric fraction of aggregate on the thermal conductivity are explored using another group of specimens: paste, mortar, and concrete (Table 3).

**Figure 1:**
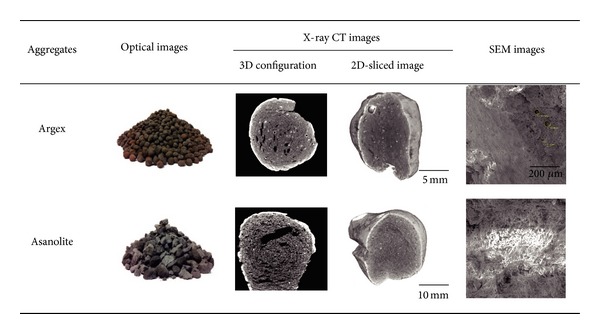
Images of lightweight coarse aggregates.

Figure 1 shows optical images of the lightweight aggregates used here. Argex contains rounded particles with randomly configured internal pores; shell-like pores exist in Asanolite; both show a noticeably great variety of pore shapes. The micrometer-scale pores observed by scanning electron microscopy corroborate the low density of the surrogate aggregates.

All specimens are subjected to slump testing and fresh density and are then cast in different molds [22]. The thermally insulated concretes are cast into specially designed thermal molds (200 mm × 200 mm × 300 mm) and three brick molds (65 mm × 114 mm × 230 mm) for the measurement of thermal conductivity. Three specimens (paste, mortar, and concrete) are cast in *Ф* 70 mm × 100 mm cylinders. All specimens are removed from the molds after 24 h and cured at room temperature and 50% relative humidity for more than 14 days. Density and compressive strength are independently measured using *Ф* 100 mm × 200 mm specimens.

### 2.3. Measurement of Thermal Conductivity

**Figure 2:**
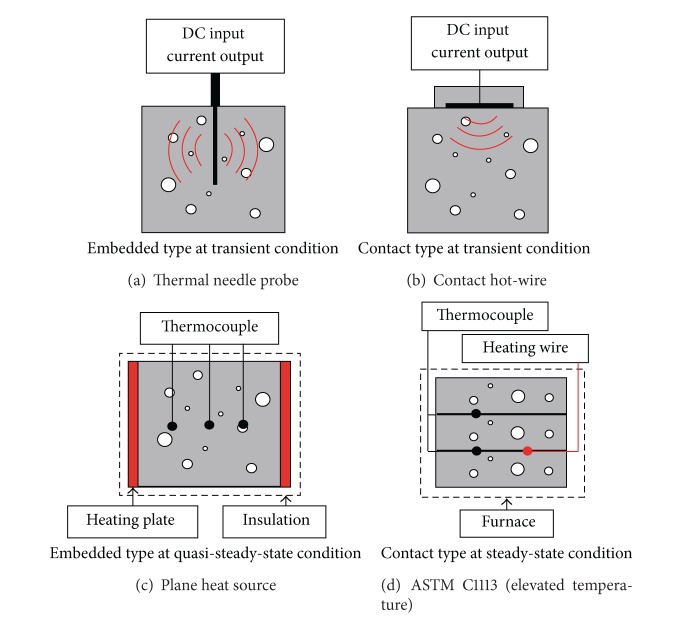
Methods for measuring thermal conductivity.

Four methods of assessing thermal conductivity are compared. They differ in the method of the heat transfer and the transducer type (Figure 2). The methods and their corresponding specimens are listed in Table 4.

#### 2.3.1. Thermal Needle Probe (Embedded Type at Transient)

The probe (stainless steel, 60 mm long, 1.3 mm diameter) contains a heating wire and thermistor (East 30 Sensors Ltd.). It is fully embedded into the specimen when it is in the thermal mold. A DC current generates the line-source heat radially from the probe, and the temperature is simultaneously monitored every 0.5 s for 3 min. The applicability of the method to concretes and other construction materials and also the detailed theory can be found elsewhere [4, 23, 24]. The probe should be embedded into the concrete before curing, limiting its usefulness regarding the *in situ* testing of concrete structures.

#### 2.3.2. Contact Hot-Wire Method (Contact Type at Transient)

The testing system (QTM-500, Kyoto Electronics Manufacturing, Co., Ltd.) follows similar principles to the thermal needle probe. However, the sensor sits on the surface of the specimen, and the line-source heat is propagated in only one direction. This method can be readily applied *in situ*, although a flat and polished contact surface is required for sound coupling.

#### 2.3.3. Planar Heat Source Method (Embedded Type at Quasi-Steady)

The heating plate provides the plane-source heat through the specimen and the sequentially embedded thermocouples detect the spatiotemporal evolution of temperature. The entire system is thermally insulated to minimize heat loss. The recorded temperature profiles are interpreted considering energy conservation based on Fourier's law. The reliability of using planar heat sources for measuring the thermal conductivity of concretes has been previously reported [4]. This method can assess relatively large specimens (with dimension of tens of centimeters), although the acquisition of a complete set of temperature profiles testing takes several days as the system approaches a steady state.

#### 2.3.4. ASTM C1113 (Contact Type at Steady)

This method was originally designed for refractories at elevated temperatures. Three brick-shaped specimens sandwich thermocouples and heating wires in between, before being heated in a furnace. First thermal equilibrium is achieved (for testing at 600°C, the soaking period for the thermal steady state takes more than 4 days). The platinum heating wire is then heated and the temperature differential measured by two thermocouples is used to compute the thermal conductivity. The coupling between the transducers and the surface of the specimen is not as complete as in the embedded testing types.

### 2.4. Test Procedures

The thermal mold designed for measurement at ambient temperature embeds two thermal needle probes and five successive thermocouples at 50 mm intervals. Once measurement using the thermal needle probe and the planar heat source is complete, the mold is dismantled, and the surface of the specimen is thoroughly cleaned and polished. Measurement using the contact hot-wire (i.e., the QTM-500 device) follows. The thermal conductivities of bricks are then independently obtained using the ASTM C1113 method at 45°C, 100°C, 200°C, 300°C, 400°C, 500°C, 600°C, 670°C, and 770°C. Measurement is repeated three times at each temperature. The furnace is heated at 55°C h^−1^. Paste, cement, and concrete specimens (*Ф* 70 mm × 100 mm cylinders) are tested using thermal needle probes. Water content and unit weights are periodically measured during curing, and conductivity values are independently assessed after 7, 14, and 28 days of curing.

### 2.5. Thermogravimetric Analysis (TGA)

Thermogravimetric analysis allows assessment of the changing proportions of calcium silicate hydrate (C–S–H) and calcium hydroxide in the hydrated cements of normal concrete during heating at 10°C min^−1^ from 25°C to 1000°C. Weight and heat flux data are obtained as the cement paste is heated. The thermal behavior is then compared with the measured thermal conductivity at elevated temperatures, allowing the elucidation of the relationship between the chemical changes in the specimens and their thermal properties.

## 3. Results and Discussion

The thermal conductivity data from the various testing methods are presented first. The reference specimens (paste, mortar, and concrete) are independently prepared to demonstrate the effects of the aggregate and the curing time. The specimens heated to ~770°C have their temperature-dependent thermal conductivities reported with the discussion of their phase change and the associated chemical reactions.

### 3.1. Thermal Conductivity

**Figure 3:**
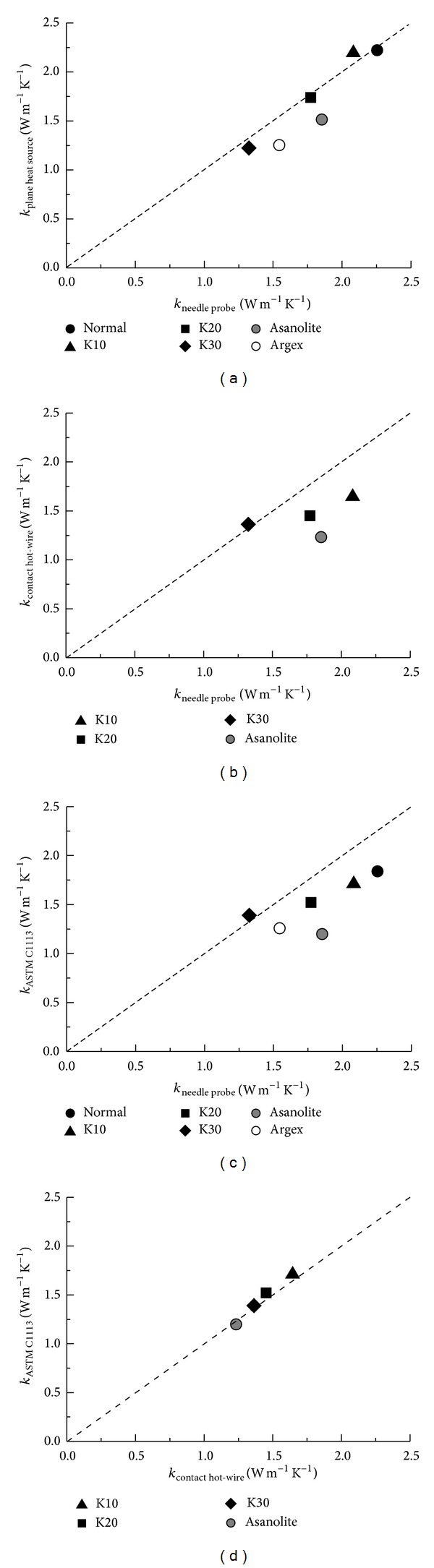
Thermal conductivity of tested specimens measured by various methods.

Figure 3 compares the measured thermal conductivities with those obtained by the thermal needle probe method. Normal concrete shows a thermal conductivity of ~2.25 W m^−1^ K^−1^; the values tend to decrease linearly with increasing fraction of glass bubbles, reaching ~1.3 W m^−1^ K^−1^ in the K30 specimen. This 42% reduction of thermal conductivity upon the addition of glass bubbles at a 30% volume fraction of the aggregates is mainly attributed to the existence of sub-micrometer-sized air voids in the glass bubbles. The density change from 2370 kg m^−3^ (normal concrete) to 2011 kg m^−3^ (K30) is accompanied by a decrease of compressive strength (from 43.9 MPa in normal concrete to 24.6 MPa in K30). The concrete specimen with Argex aggregate shows thermal conductivities of 1.25 W m^−1^ K^−1^ to 1.54 W m^−1^ K^−1^, which are lower than those of the specimen containing Asanolite. This is attributed to Argex having a lower bulk density and a higher water-adsorption capacity, which suggests that it has more internal pores than Asanolite. The air-dry densities of the specimens with Argex and Asanolite are 1848 kg m^−3^ and 1817 kg m^−3^, respectively; their respective measured compressive strengths are 37.7 MPa and 36.0 MPa. Therefore, the replacement of coarse aggregate with lightweight aggregates more efficiently reduces the density of the concrete, while minimizing the weakening of the concrete, than does the use of glass bubbles.

The testing methods with embedded probes (thermal needle and planar heat source) show similar thermal conductivity values, with less variation than the two contact-type methods, owing to the minimal heat resistance between the sensors and the tested materials (Figures 3(a), 3(b), and 3(c)). The inherent incomplete coupling of the contact hot-wire and ASTM C1113 methods leads them to underestimate thermal conductivity by ~20%; however, the two methods are consistent with each other (Figure 3(d)). The effects of the lightweight aggregates and the glass bubbles on thermal conduction are clearly represented by all the methods, but the embedded methods appear to provide quantitatively more accurate data due to the defined contact between the transducers and the specimen. The contact-type methods would likely be more applicable practically than the embedded types because the insertion of transducers is not always feasible after construction.

### 3.2. Effect of Aggregate Size

**Figure 4:**
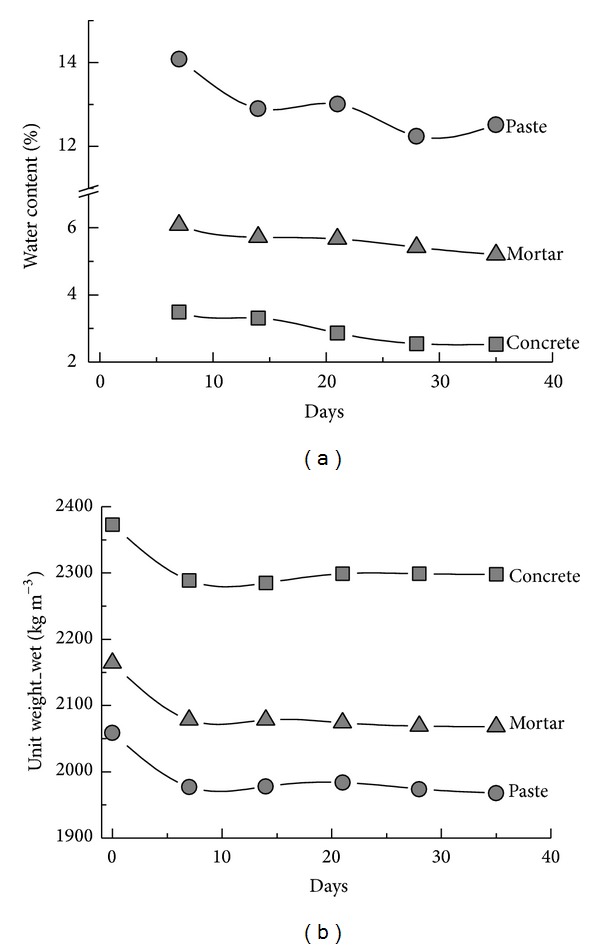
Changes in (a) water content and (b) wet unit weight of tested specimens during curing.

**Figure 5:**
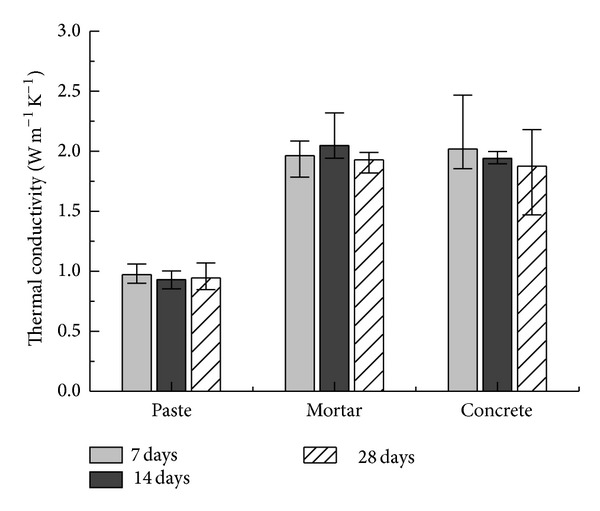
Effect of fine and coarse aggregates.

Fine and coarse aggregates are compared for their effects on the thermal conductivity of paste, mortar, and concrete specimens. The thermal needle probes are fully inserted into cylindrical specimens (Φ 70 mm × 100 mm), and conductivity is obtained after 7, 14, and 28 days of curing. Changes of unit weight and water content are also monitored (Figure 4). The paste has the highest water content and the lowest wet unit weight. Both properties decrease with time owing to water evaporation. Thermal conductivity tends to decrease slightly during curing (Figure 5), although curing appears to have a nominal effect. The paste specimen has the lowest value of ~1 W m^−1^ K^−1^; the mortar and concrete both have similar values of ~2 W m^−1^ K^−1^.

Although the presence of coarse aggregate could have facilitated heat conduction, there is no noticeable difference between the samples with coarse or fine aggregate, presumably due to the two aggregates being of similar origin, thus being similarly good thermal conductors regardless of grain size. This suggests that interfacial thermal resistance does not dominate the properties of the aggregates within the cement paste and that the volumetric fraction of aggregate in the concretes more greatly affects the thermal conduction. The water content appears to affect the thermal conduction, with the more moist paste showing lower thermal conductivity than the mortar or concrete. Figure 4 shows that the unit weight of the specimens has little effect on their thermal conductivity. Therefore, it is desirable to replace either aggregate with surrogates to reduce the thermal conductivity, provided that the specimens are not too greatly weakened.

### 3.3. Temperature-Dependent Thermal Conductivity

**Figure 6:**
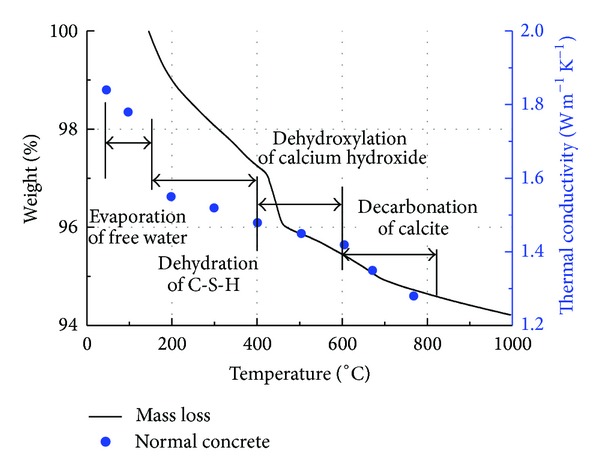
Results of thermogravimetric analysis and thermal conductivity values for normal concrete with temperature.

Figure 6 presents the results of thermogravimetric analysis of normal concrete specimens. During heating free water starts to evaporate from the cement paste at 100°C ~120°C [25]. Then, the dissociation of water linked to the C-S-H occurs between 150°C and 400°C [14, 26]; the dehydroxylation of calcium hydroxide (crystals of calcium hydroxide decompose into calcium oxide and water) follows at 400°C and 600°C, when the major weight loss and weakening of the concretes takes place [25]. The gradual reduction of weight from 600°C to 825°C is attributed to the decarbonation of calcite to calcium oxide [27]. The percentage mass losses corresponding to the dehydration of C–S–H, the dehydroxylation of calcium hydroxide, and the decarbonation of calcite are summarized in Table 5. The average thermal conductivity data for normal concrete (measured by the ASTM C1113 method, superimposed in Figure 6) gradually decrease in a way following the observed mass losses. The continuum in the hydrated cement paste appears to be lost upon heating owing to the formation of pores—which were initially occupied by microstructures such as calcium silicate hydrates and calcium hydroxide.

**Figure 7:**
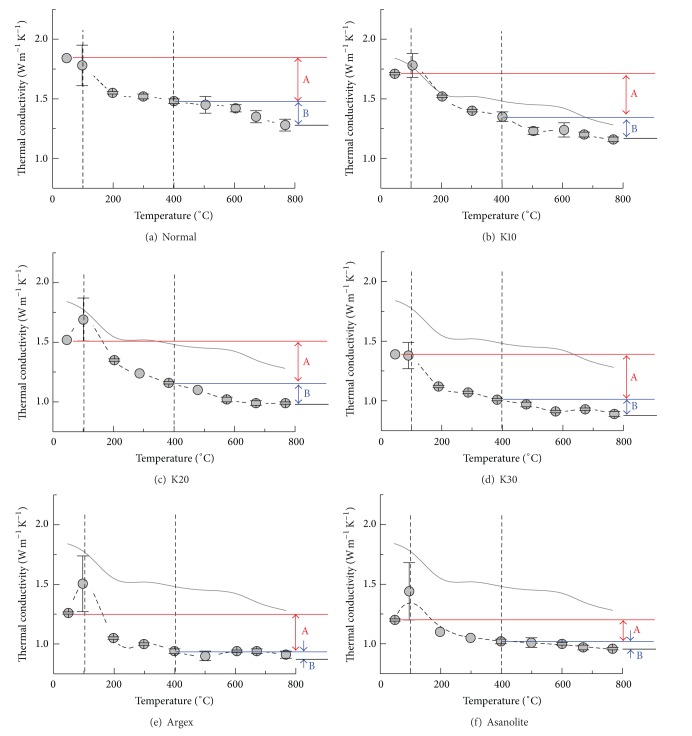
Temperature-dependent thermal conductivity of tested specimens.

Figure 7 summarizes the evolution of the thermal conductivity of the six tested specimens upon heating. The solid line denotes the behavior of normal concrete for comparison. Each specimen exhibits a sharp increase of thermal conductivity near 100°C; the pronounced variation is due to the evaporation of free water associated with the reduction of latent heat during vaporization [25, 28]. Although the formation and propagation of microcracks induced by vapor pressure after 300°C may reduce the thermal conductivity, they are not clearly manifested here. Specimens with glass bubbles exhibit major reductions of thermal conductivity by 400°C (denoted as zone A) followed by a gradual decrease (zone B). Lightweight aggregate concretes that show low thermal conductivity at ambient temperature show the greatest losses of thermal conductivity during the evaporation and dehydration phases below 400°C; quasiasymptotic behavior then follows (Figures 7(e) and 7(f)). These observations indicate that the chemical reactions at elevated temperatures do not contribute to the decrease of thermal conductivity. The presence of pores within the lightweight aggregates is likely sufficient to lessen thermal conduction and to reduce the effects of any further changes in chemical composition induced by heating. We also hypothesize that the absorption of water into the lightweight aggregates during mixing partially obstructs the dehydration of nonevaporative water from C–S–H; the subsequent chemical reactions in the lightweight aggregate concretes upon heating do not follow the analogous behavior observed in normal concretes. Nevertheless, it is evident that the type of coarse aggregate not only significantly determines the thermal conductivity at ambient temperature but also influences its behavior upon heating.

## 4. Conclusions 

The thermal behavior of thermally insulated concretes with lightweight aggregates and glass bubbles replacing the coarse aggregate normally used was characterized at ambient and elevated temperatures. An increase in the volumetric fraction of glass bubbles led the thermal conductivity of the concrete to decrease, while maintaining sufficient compressive strength for its practical use. Two lightweight aggregates were tested as replacements for coarse aggregate: their macro- and microsized pores also reduced thermal conduction in the concrete. Four methods were compared to assess the concretes. The two methods using surface-contact-type transducers (contact hot-wire method and the ASTM C1113 standard method) tended to underestimate the thermal conductivity. The presence of regular aggregate facilitated heat conduction, but the size of the aggregate was found not to affect thermal conductivity. Thermogravimetric analysis of cement pastes revealed a succession of changes of their chemical compositions during heating that followed their observed decreases of thermal conductivity. The introduction of internal pores into the specimens containing the lightweight aggregates—attributable to the thermal decomposition of their constituents upon heating—likely had a dominating effect on the thermal behavior of the concretes. This physical change had a greater effect on the thermal conductivity than did the chemical compositional changes themselves. The emergence of quasiconstant thermal conductivity above 400°C may not only be attributable to the inherently high porosity within the lightweight aggregates but also to the absorption of water into the lightweight aggregate during mixing and the delay of the dehydration of C–S–H.

## Figures and Tables

**Table 1:** Physical properties of glass bead, fine, normal, and lightweight aggregates.

Properties	Fine aggregate	Coarse aggregate			Glass bubbles
		Normal	Argex	Asanolite	
Raw material	Granite	Granite	Clay	Shale	Soda-lime borosilicate
Maximum size (mm)	1.2	25	8	19	0.065
Dry loose bulk density (kg m^−3^)	1480	1680	650	800	125
Water adsorption (%)	1.0	—	19.0	12.0	—

**Table 2:** Mix proportions.

Specimen	Cement (kg m^−3^)	Fly-ash (kg m^−3^)	Water (kg m^−3^)	Aggregates (kg m^−3^)			
				Fine	Coarse	Glass bubble	LWA
Normal	288	32	175	822	934	—	—
K10	288	32	175	870	732	12	—
K20	288	32	175	870	494	24	—
K30	288	32	175	870	227	37	—
Argex	288	32	175	834	—	—	510
Asanolite	288	32	175	834	—	—	583

**Table 3:** Mix proportions for paste, mortar, and concrete.

Mix type	W/C ratio (%)	Volume ratio (%)				Weight (kg m^−3^)			
		Cement	Water	Sand	Gravel	Cement	Water	Sand	Gravel
Paste	34.7	48.2	51.8	—	—	320	111	—	—
Mortar	54.7	17.2	29.2	53.5	—	320	175	827	—
Concrete	54.7	10.8	18.2	33.4	37.6	320	175	827	939

**Table 4:** Test methods and corresponding specimens.

Methods	Mold	Normal	K10	K20	K30	AG0	AS0
Needle probe	Thermal mold (200 mm × 200 mm × 300 mm) for ambient temperature	O	O	O	O	O	O
Plane heat source		O	O	O	O	O	O
Contact hot-wire			O	O	O		O

ASTM C1113	Brick mold (65 mm × 114 mm × 230 mm) for both ambient and elevated temperatures	O	O	O	O	O	O

**Table 5:** Mass loss measured from TGA.

Temperature range	145~400°C	400~600°C	600~825°C
Mass loss (%)	2.75	1.80	0.87
